# Pilot Results of a Digital Hypertension Self-management Program Among Adults With Excess Body Weight: Single-Arm Nonrandomized Trial

**DOI:** 10.2196/33057

**Published:** 2022-03-30

**Authors:** Folasade Wilson-Anumudu, Ryan Quan, Christian Cerrada, Jessie Juusola, Cynthia Castro Sweet, Carolyn Bradner Jasik, Michael Turken

**Affiliations:** 1 Omada Health, Inc San Francisco, CA United States; 2 Evidation Health, Inc San Mateo, CA United States

**Keywords:** hypertension, self-management, digital health, home measurement, lifestyle

## Abstract

**Background:**

Home-measured blood pressure (HMBP) in combination with comprehensive medication support and lifestyle change are the mainstays of evidence-based hypertension (HTN) management. To date, the precise components needed for effective HTN self-management programs have yet to be defined, and access to multicomponent targeted support for HTN management that include telemonitoring remain inaccessible and costly.

**Objective:**

The aim of this pilot study was to evaluate the impact of a digital HTN self-management program on blood pressure (BP) control among adults with excess body weight.

**Methods:**

A single-arm, nonrandomized trial was performed to evaluate a digital HTN self-management program that combines comprehensive lifestyle counseling with HTN education, guided HMBP, support for taking medications, and led by either a registered nurse or certified diabetes care and education specialist. A sample of 151 participants were recruited using a web-based research platform (Achievement Studies, Evidation Health Inc). The primary outcome was change in systolic BP from baseline to 3 months, and secondary outcomes included change in diastolic BP and medication adherence.

**Results:**

Participants’ mean age was 44.0 (SD 9.3) years and mean BP was 139/85 mm Hg. At follow-up, systolic and diastolic BP decreased by 7 mm Hg (*P*<.001, 95% CI –9.3 to –4.7) and 4.7 mm Hg (*P*<.001, 95% CI –6.3 to –3.2), respectively. Participants who started with baseline BP at goal remained at goal. For participants with stage 1 HTN, systolic and diastolic BP decreased by 3.6 mm Hg (*P*=.09, 95% CI –7.8 to 0.6) and 2.5 mm Hg (*P*=.03, 95% CI –4.9 to –0.3). Systolic and diastolic BP decreased by 10.3 mm Hg (*P*<.001, 95% CI –13.4 to –7.1) and 6.5 mm Hg (*P*<.001, 95% CI –8.6 to –4.4), respectively, for participants with stage 2 HTN. Medication adherence significantly improved (*P*=.02).

**Conclusions:**

This pilot study provides initial evidence that a digital HTN self-management program improves BP and medication adherence.

## Introduction

### Background

Hypertension (HTN) impacts an estimated 116.4 million or 46% of adults in the United States [1]. Annual health care costs associated with HTN are approximately US $131 billion or 3% of the US $3 trillion national health care expenditure [2]. The proportion of adults with controlled blood pressure (BP), has declined in recent years, with <44% of adults achieving this goal [3]. Poor BP control continues to be a persistent public health challenge because of the high prevalence of obesity, insufficient access to lifestyle counseling, difficulty adopting lifestyle change, medication adherence, therapeutic inertia owing to clinical uncertainty about BP data accuracy, and lack of provider access to adjust medications when needed [4-7].

Home-measured blood pressure (HMBP), when combined with more intensive human-led interventions, such as lifestyle counseling or medication titration, has shown promise in improving BP control [8]. In particular, HMBP data may be superior to in-office measurement to inform treatment decisions. The most effective interventions seem to be those that combine HMBP with medication counseling to address medication-taking beliefs and barriers and facilitate rapid medication titration [9,10]. These programs are typically staffed by pharmacists and embedded within clinics, making them difficult to scale efficiently. Clinics struggle with maximizing pharmacist time and dispensing or triaging devices.

Mobile health (mHealth) interventions can facilitate scalable, accessible, and effective management of HTN. HMBP is a core component with additional features such as automated medication reminders [11] or artificial intelligence (AI) software to provide real-time feedback on monitored BP values [12]. The bulk of innovation in this area has focused on fully automated solutions, but these interventions have had marginal clinical efficacy and have not been shown to be more clinically effective than HMBP alone. Few programs combine HMBP with access to what is known to work—human-led support that interprets BP data, supports lifestyle change, and titrates medication.

Self-management is a daily process where individuals actively engage in the management of a chronic illness, and structured programs such as Diabetes Self-Management Education and Support have demonstrated broad clinical efficacy [13]. Similarly, for HTN, supported self-management interventions, which are complex and employ a broad range of support strategies, have been found to improve BP control [14]. While robust evidence is still lacking, early evidence suggests that mHealth interventions with more comprehensive features are likely to be more effective [15,16]. The self-management approach places focus on the individual to play a critical role in their care in collaboration with health care providers [17].

### Objective

The goal of this pilot study was to evaluate the impact on BP control of a registered nurse (RN) or certified diabetes care and education specialist (CDCES)-led mHealth HTN self-management program that combines comprehensive lifestyle counseling (for dietary changes, weight loss, physical activity, and stress management) with HTN education, guided HMBP, support for taking medications, and social support, among individuals with uncontrolled HTN.

## Methods

### Participants

Members of a web-based health community (Achievement, Evidation Health Inc) were invited to participate in this study. Achievement is a web- and mobile-based community in the United States where members can connect their activity trackers and fitness and health apps to the platform and, by logging activities, accumulate points that are redeemable for monetary rewards. Additionally, members self-report on various health conditions and are invited to participate in remote research opportunities as relevant studies come available. In this study, recruitment was targeted to members who had self-reported an HTN diagnosis. Invited members were linked to a web-based research study platform (Achievement Studies, Evidation Health Inc) where study eligibility was assessed using automated screener questions. Eligibility criteria for the pilot study included the following: being a US resident, being at least 18 years of age, self-reported HTN diagnosis, self-reported recent systolic BP (SBP) measurement of ≥130 mm Hg or diastolic BP (DBP) of ≥80 mm Hg within 1 month prior to screening, a BMI of ≥25 kg/m^2^ (≥23 kg/m^2^ if they self-identified as Asian), and access to a computer or smartphone to participate in the virtual HTN program.

### Procedures

If eligible after completing the web-based screener, potential participants were asked to sign an electronic informed consent form and complete a web-based baseline survey, consisting of questions about their demographics, health and HTN history, HTN medication usage, and patient-reported outcomes (as described in *Measurements*). After completion of the baseline survey, potential participants were instructed to set up a HTN program account. After completion of both the baseline survey and program account setup, participants were considered to have been enrolled in the study. In the first week of the program, participants were instructed to take BP readings and were asked to take multiple readings throughout the week. The average across all readings in the first 30 days was used as the baseline measurement for the pilot study.

Throughout the study period, participants were encouraged to engage with the supported mHealth HTN self-management program at the time and frequency of their choosing. After 3 months of study enrollment, participants were asked to complete a final web-based survey and submit BP measurements throughout the last week of the program. Study participation was completely remotely, and participants received a small electronic gift card compensation for completion of all data collection.

### Ethical Considerations

This study was exempted from ethics approval by the Western International Review Board (WIRB Work Order #1-1249356-1). All participants in this study provided informed consent.

### Measurements

All study measures were collected at baseline and at 3 months. BP was collected using a cellular-connected BP monitor (BodyTrace Inc) that was provided to every participant as a core component of the program. Participants were provided with instructions on how to take accurate resting BP readings [18]. Participants were asked to submit a BP measurement at baseline and at 3 months. Baseline BP was defined as the average of at least 3 BP readings taken across 2 days in a 30-day measurement window closest to the baseline time point. Similarly, final BP was defined as the average of at least 3 BP readings taken across 2 days in a 30-day measurement window closest to the 3-month time point. Throughout the study, participants were encouraged to submit additional BP measurements in accordance with the schedule recommended by their health care professional. Program participants were also provided with a cellular-connected weight scale (BodyTrace Inc) and were asked to weigh in daily throughout the program. The median weight collected during weeks 1 and 12 of the program was used as weight outcomes for the pilot study.

The following patient-reported outcomes were assessed through a web-based survey administered at baseline and during week 12 of the program: the Consumer Health Activation Index (CHAI), a 10-item scale measuring health-related activation and engagement [19], with scores between 0 and 79 reflect low activation, scores between 80 and 94 reflecting moderate activation, and 95 and 100 reflecting high activation [19]; the Self-Efficacy for Managing Chronic Disease scale, 6 items on a scale of 1 (not at all confident) to 10 (totally confident) measuring one’s confidence in doing certain activities that are common across many chronic conditions, with higher scores indicating higher self-efficacy [20]; HTN medication usage (ie, a self-report of medications, dosage, and timing of administration); and the Simplified Medication Adherence Questionnaire (SMAQ), a 6-item scale that categorizes respondents as adherent or nonadherent on the basis of recent patterns of medication-taking behaviors [21].

### Intervention

The Omada for Hypertension Program is a supported digital HTN self-management program that is commercially available through business-to-business relationships with organizations that pay to make the service available to their members (typically employees or health plan members). The program can be accessed through mobile devices (ie, smartphones or tablet devices) or PCs. The program offers a HTN education curriculum along with comprehensive lifestyle self-management support, including support for weight loss, dietary changes (aligning with the Dietary Approaches to Stop Hypertension and Mediterranean Diets), increased physical activity, support for medication adherence, social support, and a cellularly connected BP monitor and body weight scale. Participants are assigned to a HTN specialist coach (either a RN or CDCES), who supports their progress longitudinally through the program, addressing specific questions, providing feedback on HMBP data, supporting medication-taking, and helping prepare participants for primary care physician and specialist visits. Data from the BP monitor and weight scale are provided back to the participant in their program account; the data are also used by the HTN specialist coach to provide counseling on dietary changes, changes in physical activity, and to encourage communication with their health care providers when timely medication adjustments may be needed. Participants are also placed in a virtual peer group and can communicate with other program users through a secure group discussion board.

### Statistical Analysis

The study was powered to detect a clinically meaningful 4 mm Hg reduction in the primary outcome of resting SBP. With an estimated SD of 12 mm Hg and power set to 90%, the minimum sample size needed was 113. To allow for a potential 10% loss to follow-up of study participants at 3 months, and an estimate of 30% of participants’ resting SBP being <130 mm Hg at baseline, a total of 150 participants were planned for study enrollment.

Descriptive statistics (eg, means and frequencies) were performed to describe the demographic and baseline clinical characteristics of the enrolled study population. Correlations (Pearson correlation for continuous variables and Spearman correlation for categorical variables) were run to detect potential baseline confounders (age, gender, and BMI) of resting SBP and DBP; no significant correlations were found. Two-tailed paired *t* tests were used to assess significant changes in BP measurements, weight, and patient-reported outcomes from baseline to follow-up. The definitions of stage 1 and stage 2 HTN differ in accordance with BP measurement methodology, with the HMBP threshold for stage 2 HTN (135/85 mm Hg) being lower than the office-based threshold (140/90 mm Hg) [22]. While the HMBP threshold is the most pertinent for this study given the BP measurement methodology, the majority of trials have traditionally used office-based thresholds. Hence, for BP post hoc analyses, participants were stratified by HTN stage using both home-based and in-office cutoffs for stage 1 and stage 2 HTN, and paired *t* tests were performed within these groups to examine changes in BP on the basis of the baseline BP stage.

The McNemar nonparametric test was used to examine the change in the proportion of the study population that was adherent to their medication regimen from baseline to follow-up and 2-tailed paired *t* tests were used to assess change in the number of HTN medications used by participants.

Outcomes were analyzed using both intention-to-treat analysis (with baseline carry forward of missing data) and complete case analysis (among those with complete baseline and follow-up data). The outcomes were similar in magnitude and statistical significance using both analytic methods, and thus we present the results on the sample of study participants with complete data from both timepoints.

## Results

### Study Recruitment

**Figure 1:**
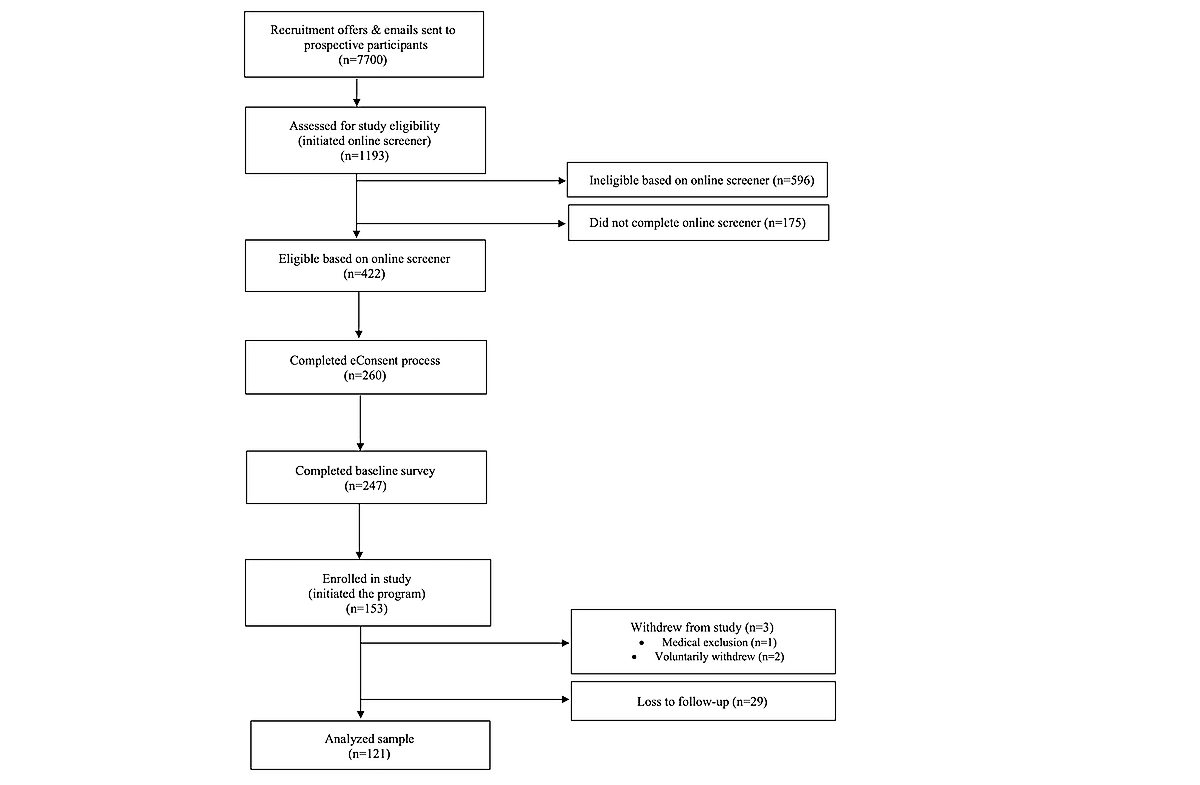
Flow diagram of study participants through each stage of the study.

The final enrolled sample comprised 153 participants. Three participants were withdrawn from the study: one developed a medical condition that precluded participation and 2 requested to voluntarily withdraw from the study. At follow-up, 80% (n=121) of participants had a complete follow-up BP measurement, and 81% completed the web-based questionnaire; 29 (19%) participants were lost to follow-up. Figure 1 outlines the flow of participants through each stage of the study. The analyzed sample (n=121) and those lost to follow-up (n=30) were compared for differences in baseline characteristics, which might impact the BP outcomes. There were no significant differences in baseline SBP, weight, medication adherence, gender, age, or race and ethnicity. However, baseline DBP was slightly higher in the group lost to follow-up than in those who stayed in the study (88 vs 84 mm Hg).

### Participant Characteristics at Baseline

Table 1 shows the baseline characteristics of the study participants, including missing data for participants who did not complete various metrics at baseline. The sample was 56% female, 72% White, and 11% of participants self-identifying as African American. The baseline mean SBP for the sample was 139 mm Hg and mean DBP 85 was mm Hg. In total, 76% of participants reported taking medication for HTN medication at the start of the program and 27% self-reported being adherent to their medication regimen (SMAQ).

The average weight of the sample at program start was 224 lbs. The mean CHAI health activation score at baseline was 71.9 (SD 14.2). The mean self-efficacy score at baseline was 44.3 (SD 11.7) out of a possible 60.

**Table 1:** Baseline participant characteristics (N=151).

Baseline characteristics^a^	Values
Age (years), mean (SD)	44.0 (9.3)
Females, n (%)	85 (56.3)
White participants, n (%)	109 (72.2)
Black or African American participants, n (%)	17 (11.3)
Systolic blood pressure (mm Hg; n=148), mean (SD)	138.5 (13.1)
Diastolic blood pressure (mm Hg; n=148), mean (SD)	84.6 (8.9)
Weight (lbs; n=149), mean (SD)	223.7 (52.0)
Patient activation (n=144), mean (SD)	71.9 (14.2)
Self-efficacy (n=144), mean (SD)	44.3 (11.7)
Taking hypertension medication, n (%)	114 (75.5)
Adherent to current medications, n (%)	41 (27.2)

^a^The table includes missing data from participants who did not complete baseline data collection.

### Program Engagement

Participants used their BP cuff an average of 7.2 times per week across the 12 weeks of the program. Participants weighed in an average of 4.7 times per week, interacted with their coaches an average of 1.2 times per week, completed an average of 0.75 lessons per week, interacted with their peer groups an average of 0.6 times per week, tracked their physical activity 5.4 times per week, and tracked meals an average of 9.0 times per week.

### BP Outcomes

In Table 2, the baseline and follow-up changes in mean BP are listed for the analyzed sample (n=121). Baseline SBP significantly decreased by an average of 7 mm Hg (*t*_120_=–6.0; *P*<.001; 95% CI –9.3 to –4.7). DBP significantly declined by 4.7 mm Hg (*t*_120_=–6.1; *P*<.001; 95% CI –6.3 to –3.2). Weight also decreased by an average of –2.4 lbs (*t*_119_=–3.5; *P*<.001; 95% CI –3.7 to –1.0), with 14% of the sample losing 5% of their initial body weight. Table 3 presents changes in BP by baseline BP clinical category based on home-based versus in-office thresholds for stage 2 HTN [22]. All study participants who started with BP at goal remained at goal (26/121, 21.5%). For participants with stage 1 HTN at baseline (SBP 130-134 mm Hg, DBP 80-84 mm Hg), there was a trend toward significant reduction in SBP by 3.6 mm Hg (*t*_18_=–1.8; *P*=.09; 95% CI –7.8 to 0.6), and DBP significantly decreased by 2.6 mm Hg (*t*_18_=–2.4; *P*=.03; 95% CI –4.9 to –0.3). BP reduction was greater for those with stage 2 HTN at baseline (SBP≥135 mm Hg or DBP≥85 mm Hg), with a 10.3 mm Hg reduction in SBP (*t*_75_=–6.5; *P*<.001; 95% CI –13.4 to –7.1) and a 6.5 mm Hg reduction in DBP (*t*_75_=–6.1; *P*<.001; 95% CI –8.6 to –4.4). Using in-office range thresholds, those with stage 1 HTN (SBP 130-139 mm Hg, DBP 80-89 mm Hg) had a significantly decreased DBP by 2.9 mm Hg (*t*_38_=–3.1; *P*<.004; 95% CI –4.8 to –0.98). For stage 2 HTN defined using office ranges (SBP≥140 mm Hg or DBP≥90 mm Hg), SBP reduction was 13.4 mm Hg (*t*_55_=–7.3; *P*<.001; 95% CI –17.1 to –9.7) and a 7.7 mm Hg reduction in DBP (*t*_55_=–5.8; *P*<.001; 95% CI –10.4 to –5.1).

**Table 2:** Baseline to follow-up changes in outcomes in participants with complete data.

Parameters	Participants, n	Baseline value	Follow-up value	Difference	*t* test (*df*)	95% CI	*P* value
Systolic blood pressure (mm Hg)	121	137.7	130.7	–7.0	–6.0 (120)	–9.3 to –4.7	<.001
Diastolic blood pressure (mm Hg)	121	83.9	79.1	–4.7	–6.1 (120)	–6.3 to –3.2	<.001
Weight (lbs)	120	221.8	219.4	–2.4	–3.5 (119)	–3.7 to –1.0	.001
Patient activation	115	70.9	74.0	3.1	2.4 (114)	0.6 to 5.6	.02
Self-efficacy	115	43.9	44.4	0.4	0.5 (114)	–1.5 to 2.3	.65

**Table 3:** Baseline to follow-up changes in blood pressure by the baseline blood pressure category from among participants with complete data.

Parameters			Participants, n		Baseline value, mm Hg		Follow-up value, mm Hg		Difference, mm Hg		*t* test (*df*)		95% CI		*P* value				
**Home range**																			
	**Systolic blood pressure**																		
		Blood pressure at goal		26		122.0		122.0		0.0		0.0 (25)		–3.4 to 3.4		1.00			
		Stage 1 hypertension		19		130.0		126.0		–3.6		–1.8 (18)		–7.8 to 0.6		.09			
		Stage 2 hypertension		76		145.0		135.0		–10.3		–6.5 (75)		–13.4 to –7.1		<.001			
	**Diastolic blood pressure**																		
		Blood pressure at goal		26		74.0		73.0		–1.1		–0.9 (25)		–3.7 to 1.5		.40			
		Stage 1 hypertension		19		79.0		76.4		–2.6		–2.4 (18)		–4.9 to –0.3		.03			
		Stage 2 hypertension		76		88.4		81.9		–6.5		–6.1 (75)		–8.6 to –4.4		<.001			
**Office range**																			
	**Systolic blood pressure**																		
		Blood pressure at goal		26		122.0		122.0		0.0		0.0 (25)		–3.4 to 3.4		1.00			
		Stage 1 hypertension		39		133.0		130.0		–2.5		–1.7 (38)		–5.4 to 0.5		.10			
		Stage 2 hypertension		56		148.0		135.0		–13.4		–7.3 (55)		–17.1 to –9.7		<.001			
	**Diastolic blood pressure**																		
		Blood pressure at goal		26		74.0		73.0		–1.1		–0.9 (25)		–3.7 to 1.5		.40			
		Stage 1 hypertension		39		81.4		78.5		–2.9		–3.1 (38)		–4.8 to –0.98		.004			
		Stage 2 hypertension		56		90.1		82.4		–7.7		–5.8 (55)		–10.4 to –5.1		<.001			

### Medication Outcomes

Table 4 presents changes in medication status and medication adherence from baseline to follow-up. Medication adherence, as measured by the SMAQ, improved for the total sample by 14.4% from 36.7% to 51.1% (McNemar *χ*^2^_1,90_=5.8, *P*=.02). When we conducted post hoc analyses by HTN stage, there were no significant changes in the number of medications among those who began the program at goal or with stage 1 HTN. However, the number of HTN medications increased from 1.0 to 1.2 (*t*_76_=2.2; *P*=.03; 95% CI 0.01-0.30) among those with baseline stage 2 HTN based on the home-based thresholds.

**Table 4:** Baseline to follow-up changes in medication status.

Parameters			Participants, n		Baseline value		Follow-up value		Difference		*t* test (df)		McNemar *χ*^2^ (df)		95% CI		*P* value
**Total sample**																	
	Average number of hypertension medications	117		1.1		1.2		0.1		1.1 (116)		N/A^a^		–0.05 to 0.17		.28	
	Adherent to medications (Simplified Medication Adherence Questionnaire), %	90^b^		36.7		51.1		14.4		N/A		5.8 (90)		N/A		.02	
**Stage 2 subsample**																	
	Average number of hypertension medications	77		1.0		1.2		0.2		2.2 (76)		N/A		0.01 to 0.30		.03	
	Adherent to medications (Simplified Medication Adherence Questionnaire), %	54^b^		35.2		46.3		11.1		N/A		2.0 (54)		N/A		.16	

^a^N/A: not applicable.

^b^Among participants taking ≥1 hypertension medications at baseline.

### Patient-Reported Outcomes

Patient activation on the CHAI significantly increased by an average of 3.1 points (*t*_114_=2.4; *P*=.02; 95% CI 0.6-5.6) but self-efficacy did not improve significantly (*P*=.65).

## Discussion

### Principal Findings

The results of this pilot study provide initial evidence that a comprehensive, human-led digital HTN self-management program that includes lifestyle support, medication adherence, and guided HMBP is associated with improved BP control, weight, and medication adherence in a sample of individuals with uncontrolled HTN. Furthermore, those who started the program with stage 2 HTN achieved the greatest improvement in BP control with an average change of 10.3 mm Hg and 6.5 mm Hg in SBP and DBP, respectively. Those who started the program with their BP at goal remained at goal at the end of the study. This pilot study was successful in detecting significant weight loss and improvement in medication adherence. Program engagement was strong, as shown by the high frequency of use across the various features of the digital platform.

The findings from this pilot study are consistent with those of prior studies on digital HTN programs that showed improvements in BP control, weight, and medication adherence [11,16,23,24]. While most participants saw significant improvements in their BP, those with stage 2 HTN at baseline saw the greatest improvement, with a magnitude of decrease similar to those seen with interventions that are led by pharmacists or nurse practitioners [9]. Additionally, the magnitude of BP reduction observed in this program is comparable to that of previous studies [25,26].

There were no significant changes seen in self-efficacy; this may be in part owing to participants reporting relatively high self-efficacy at baseline. There were significant improvements in patient activation, which is arguably more comprehensive and encompassing than self-efficacy for managing HTN [27].

### Limitations

The single-arm, nonrandomized design of this pilot study harbors the challenge of unknown causal inference; thus, future studies with a comparison or control group are needed to confirm the results. Second, all measurements were collected at home using cellularly connected devices. While there is a risk of small intradevice measurement variability, participants did use the same device for their measurements across the study, so any measurement error is likely to be systematic within individuals. Third, participants self-selected from the web-based health community into the research opportunity; therefore, it is possible that the study population recruited for the pilot study is not fully representative or generalizable of the population of individuals living with HTN in the United States. The pilot study was conducted from February to July 2020 during the first wave of COVID-19 exposure. This may have influenced participants’ ability to fully engage in the program in the midst of unprecedented stressors and disruptions in health care. However, the observation of significant clinical benefits during this time is an encouraging and suggests that the results might have been stronger if the study were conducted under nonpandemic conditions.

Finally, the pilot study length was brief (3 months); while this is sufficient time to detect meaningful BP changes, it is a short time frame in which to produce clinically meaningful weight loss, which may explain the modest weight loss achieved by study participants. Finally, the size of the study sample limited the ability to conduct meaningful subgroup analyses and limited the statistical power for analyses of secondary and tertiary outcomes.

### Conclusions

The COVID-19 pandemic has created unexpected opportunities for digital health, with more routine care, including care for chronic conditions, transitioning to remote delivery, greater demand emerging for remotely delivered solutions, and reconsideration of regulations that previously slowed growth and scalability [28]. HTN self-management is an obvious fit for digital health solutions, and the results of this pilot study add to the growing body of evidence that human-supported digital self-management programs can improve outcomes for those with chronic conditions [29]. With proper design, the essential features of supported HTN self-management, including comprehensive counseling for lifestyle changes, HMBP with actionable feedback, and medication taking support, can be effectively translated to a digital format and result in strong program engagement, improved activation, and desirable clinical outcomes for people with HTN. Future research on this program will focus on the sustainability of the clinical outcomes, the robustness of the clinical benefit under increasingly rigorous testing conditions, and research among more diverse populations to promote health equity from new digital health solutions.

## Abbreviations

BP: blood pressure
CDCES: certified diabetes care and education specialist
CHAI: Consumer Health Activation Index
DBP: diastolic blood pressure
HMBP: home-measured blood pressure
HTN: hypertension
mHealth: mobile health
RN: registered nurse
SBP: systolic blood pressure
SMAQ: Simplified Medication Adherence Questionnaire
